# Comparative Study of T-Cell Repertoires after COVID-19 Immunization with Homologous or Heterologous Vaccine Booster

**DOI:** 10.3390/pathogens13040284

**Published:** 2024-03-27

**Authors:** Elizabeth-Barbara Tatsi, Filippos Filippatos, Thomas Bello, Vasiliki Syriopoulou, Athanasios Michos

**Affiliations:** 1 Infectious Diseases and Chemotherapy Research Laboratory, First Department of Pediatrics, Medical School, “Aghia Sophia” Children’s Hospital, National and Kapodistrian University of Athens, 11527 Athens, Greece; etatsi@med.uoa.gr (E.-B.T.); filippat@med.uoa.gr (F.F.);; 2 Adaptive Biotechnologies, Seattle 98109, WA, USA; tbello@adaptivebiotech.com

**Keywords:** SARS-CoV-2, COVID-19, Ad26.COV2.S, BNT162b2, immunity, vaccination

## Abstract

Sequencing of the T-cell repertoire is an innovative method to assess the cellular responses after immunization. The purpose of this study was to compare T-cell repertoires after COVID-19 immunization with homologous (HOB) and heterologous (HEB) boosting. The study included 20 participants with a median age of 27.5 (IQR:23) years, who were vaccinated with one dose of the Ad26.COV2.S vaccine and were boosted with either Ad26.COV2.S (*n* = 10) or BNT162b2 (*n* = 10) vaccine. Analysis of the T-cell receptor beta locus (TCRβ) sequencing one month after the booster dose identified that the HEB compared to the HOB group exhibited a higher number of both total and COVID-19-related functional T-cell rearrangements [mean of total productive rearrangements (TPRs): 63151.8 (SD ± 18441.5) vs. 34915.4 (SD ± 11121.6), *p* = 0.001 and COVID-19–TPRs: 522.5 (SD ± 178.0) vs. 298.3 (SD ± 101.1), *p* = 0.003]. A comparison between the HOB and HEB groups detected no statistically significant differences regarding T-cell Simpson clonality [0.021 (IQR:0.014) vs. 0.019 (IQR:0.007)], richness [8734.5 (IQR:973.3) vs. 8724 (IQR:383.7)] and T-cell fraction [0.19 (IQR:0.08) vs. 0.18 (IQR:0.08)]. HEB also exhibited a substantially elevated humoral immune response one month after the booster dose compared to HOB [median antibody titer (IQR): 10115.0 U/mL (6993.0) vs. 1781.0 U/mL (1314.0), *p* = 0.001]. T-cell repertoire sequencing indicated that HEB had increased SARS-CoV-2-related T-cell rearrangements, which was in accordance with higher humoral responses and possibly conferring longer protection. Data from the present study indicate that the administration of different COVID-19 vaccines as a booster may provide better protection.

## 1. Introduction

The human coronavirus disease 2019 (COVID-19), which is caused by the SARS-CoV-2 virus, emerged in late 2019 and continues to cause significant morbidity and mortality. To combat the pandemic triggered by the virus, vaccines based on different platforms were quickly manufactured using novel technologies. COVID-19 vaccines that were manufactured based on mRNA technology like the BNT162b2 vaccine or the recombinant, replication-incompetent adenovirus vector Ad26.COV2.S were widely used during the pandemic [1].

Both vaccines contain the SARS-CoV-2 spike protein, which is critical for the virus to attach and enter human cells by binding to the human receptor angiotensin-converting enzyme 2 (ACE2) through the virus receptor binding domain (RBD) of the S1 subunit [2,3,4].

Laboratory studies for the evaluation of vaccines’ B-cell immune responses after COVID-19 immunization focused on the levels and kinetics of anti-spike and neutralizing antibodies [5,6]. However, studies on cellular immune responses are also essential for the investigation of long-term vaccine effectiveness [7,8,9]. Several conventional laboratory methods have been used to estimate the T-cell response against SARS-CoV-2, like enzyme-linked immunosorbent assay (ELISA) methods for interferon-γ release measurement and flow cytometry [7,10]. Novel methods like the sequencing of T-cell receptors (TCRs) can provide valuable information regarding the rearrangements, richness and clonality of T-cell repertoires [11,12].

TCRs are located on the surface of T lymphocytes. Their role is to recognize the antigen–major histocompatibility complexes [13]. The developed antigen specific TCRs are dimers consisting of the chain’s alpha and beta or gamma and delta. TCR chains include the following segments: variable (V), joining (J), diversity (D) and constant (C). Rearrangements of V and J segments provide a wide range of antigen recognition. Specifically, the specificity of TCRs to antigens derived from the sequence of the hypervariable complementarity-determining region 3 (CDR3) in the V gene [14]. However, the breadth of these rearrangements and their association with different infectious diseases remains to be elucidated [15].

To date, limited studies have investigated the T-cell response after the administration of different vaccines using the sequencing methodology of the T-cell repertoire and mostly in combination with flow cytometry. The first study was in 2015, in which nine volunteers vaccinated with YF-17D for yellow fever virus participated. They detected approximately 2000 different clones of T-cells 14 days after vaccination, of which only 5–6% remain detectable 90 days post-vaccination [16]. In 2017, Herati et al. studied the T-cell response to the influenza vaccine in 12 individuals 0 and 7 days after vaccination using the sequencing of TCRs. They identified the oligoclonal response of T-helper CD4^+^ cells to influenza vaccine, that was maintained with annual vaccination [17].

Regarding the evaluation of SARS-CoV-2 vaccines using TCR sequencing, limited studies have been published. Alter et al. investigated the efficacy of the Ad26.COV2.S COVID-19 vaccine against different SARS-CoV-2 variants (Wild type, B.1.1.7, CAL.20C, P.1 and B.1.351) in 20 individuals [18]. Another study compared the immune responses in individuals with or without previous SARS-CoV-2 infection after SARS-CoV-2 mRNA immunization. They found that individuals with hybrid immunity produced a new antigen-specific repertoire [19]. Yin et al. investigated the immune response of influenza and SARS-CoV-2 immunization in mice. They found that the vaccination with a Toll-like receptor 7 (TLR7) agonist nanoparticle adjuvanted influenza subunit vaccine was effective against different influenza strains and in combination with a SARS-CoV-2 subunit vaccine enhanced the immune response against variants of both viruses [20].

The aim of this study was to compare T-cell repertoires and the humoral response after COVID-19 immunization with homologous (HOB) and heterologous (HEB) boosting using T-cell receptor beta locus deep sequencing. 

## 2. Materials and Methods

### 2.1. Study Design and Participants

This prospective cohort study enrolled volunteer adults who were vaccinated with one dose of the Ad26.COV2.S COVID-19 vaccine from June to August 2021 and were boosted with either a homologous (Ad26.COV2.S vaccine; Janssen Pharmaceuticals, Beerse, Belgium) or heterologous (BNT162b2 vaccine; Pfizer, New York, NY, USA and BioNTech, Mainz, Germany) dose from January to March 2022. The participants who came for the booster COVID-19 vaccination at the vaccination center of the “Aghia Sophia” hospital were asked to participate in the study.

Blood sampling was performed one month after the administration of the booster dose. Specifically, 3 mL of blood samples were obtained in vacuum tubes with a gel and clot activator, specific for serum separation (Weihai Sunway Medical Technology, Petaling Jaya, Selangor, Malaysia) and were kept at room temperature to form a clot for 20–30 min. Serum samples were collected after centrifugation at 2000 rpm for 10 min. Serum samples were stored at −20 °C until analysis. 

Each participant completed a form containing demographic and clinical data, as well as adverse events (AEs) after each dose. Data included sex, age, smoking, body mass index (BMI; underweight ≤ 18.5 kg/m^2^, normal weight = 18.5–24.9 kg/m^2^, overweight = 25–29.9 kg/m^2^ and obesity ≥ 30 kg/m^2^), history of underlying diseases (cardiac, pulmonary, hyperlipidemia and hypertension, Type 1 diabetes mellitus, Hashimoto’s thyroiditis, autoimmune hepatitis, etc.), allergies (to any kind of allergen and type of manifestation) and SARS-CoV-2 infection. Local AEs including pain, edema, pruritus, erythema, or systemic AEs such as fatigue, fever, headache, myalgias, etc., were also recorded after each vaccine dose.

The study was conducted in the Infectious Diseases Laboratory, First Department of Pediatrics, Medical School, “Aghia Sophia” Children’s Hospital, National and Kapodistrian University of Athens, and the protocol was approved by the scientific and bioethics committee of “Aghia Sophia” Children’s Hospital (No. 3585 and 9643) and informed consent was obtained from all participants.

### 2.2. Total Antibody Detection against SARS-CoV-2

To detect antibodies against SARS-CoV-2 spike protein developed by vaccination, serum samples were tested using the Elecsys Anti-SARS-CoV-2 S (Roche Diagnostics, Basel, Switzerland) reagent on a Cobas e 411 immunoassay analyzer according to the manufacturer’s instructions. This reagent detects total antibodies (TAbs-S: IgA, IgM and IgG) against the S1 subunit of the wild type SARS-CoV-2 spike protein. 

To verify the absence of a breakthrough or previous COVID-19 infection one month after the booster dose, serums were also tested with the Elecsys Anti-SARS-CoV-2 (Roche Diagnostics, Basel, Switzerland) reagent on the Cobas e 411 immunoassay analyzer for the semiquantitative detection of the total antibodies (TAbs-N: IgA, IgM and IgG) against wild type SARS-CoV-2 nucleocapsid (N) protein. The viral nucleocapsid protein is not included in the vaccines; thus, the anti-N antibody detection (TAbs-N) indicates SARS-CoV-2 infection. 

Both assays are electrochemiluminescence immunoassays (ECLIA), which are based on a double-antigen sandwich ELISA methodology. The values of TAbs-S ≥ 0.8 U/mL and TAbs-N ≥ 1 cut-off-index (COI) are positive.

### 2.3. T-Cell Receptor Beta Sequencing and Bioinformatic Analysis

DNA was extracted from 200 μL peripheral whole blood employing the QIAamp DNA Blood Mini Kit (Qiagen, Hilden, Germany) according to the manufacturer’s instructions. Deep quantitative targeted next generation sequencing (NGS) of the human T-cell receptor beta (TCRβ) locus on chromosome 7q34—including the V gene, CDR3 region, N1 junction, D gene, N2 junction and J gene—was performed using adaptive immunosequencing (Adaptive Biotechnologies, Seattle, WA, USA) as previously described [14,21,22] with data retrieved from the ImmunoSEQ Analyzer software. 

For the bioinformatic analysis, three general TCR metrics reflecting the immune competency were used. Simpson clonality and downsampled rearrangements were used to describe the overall evenness and richness of the TCR repertoire, respectively, which is robust to sequencing depth. The T-cell fraction quantifies the percentage of T-cells amongst all nucleated cells in the periphery [23,24,25].

More specifically, Simpson clonality is a diversity metric ranging from zero (polyclonal) to one (monoclonal) that quantifies the evenness of clone frequencies in a repertoire. It is calculated as $\;\sqrt{\sum p_{i}^{2}}$, where $p_{i}\;$is the proportional abundance of productive clone i. Repertoire richness (or downsampled rearrangements) is quantified by counting the number of unique rearrangements (TCRβ CDR3 nucleotide sequences, clonotypes) observed after computationally downsampling to a common number of templates (*n* = 10,000) to adjust for template count. The T-cell fraction is calculated as the total number of T-cells detected divided by the total number of nucleated cells detected in a sample, both measured via the bias-controlled PCR assay. 

A set of approximately 160,000 high-confidence SARS-CoV-2-associated TCRβ sequences from patients’ samples were retrieved from the immuneACCESS website (https://clients.adaptivebiotech.com/immuneaccess; accessed on 15 December 2023, Adaptive Biotechnologies, Seattle, WA, USA). These sequences were compared to those of the present study to quantify the number and proportional abundance of COVID-reactive clones in each repertoire using the immunoSEQ T-MAP™ COVID Search Tool available through the immunoSEQ Analyzer (Adaptive Biotechnologies, Seattle, WA, USA). Hence, the immunoSEQ T-MAP™ COVID Search Tool was used to map the identified the productive (functional) TCRβ rearrangements to the SARS-CoV-2 genome according to the ImmuneCODE database [18,26,27].

### 2.4. Statistical Methods

Absolute and relative frequencies (%) were used to describe the qualitative variables such as demographic characteristics, while mean, standard deviation (SD), median and interquartile range (IQR) were used for quantitative data. Differences between independent samples were assessed via an uncorrected Mann–Whitney U test. Spearman’s rho (r) correlation coefficient was used for associations between continuous variables. The assumption of normality was checked through kurtosis and skewness and Shapiro–Wilk tests. The statistical significance level was set as a *p*-value ≤ 0.05. Statistical analysis was performed using R version 4.2 (R Core Team, 2022, Vienna, Austria) or SPSS version 26.0 (IBM Corp., released 2019. IBM SPSS Statistics for Windows, Version 26.0. Armonk, NY, USA: IBM Corp).

## 3. Results

### 3.1. Study Population and Reactogenicity

A total of 20 participants vaccinated with one dose of the Ad26.COV2.S COVID-19 vaccine were included in the study. Their median age was 27.5 (IQR: 23) years and 55% (11/20) were males. Their median BMI was 23.6 (IQR: 4.5) kg/m^2^, 30% (6/20) were smokers, 45% (9/20) had allergies and 25% (5/20) had an underlying disease. Ten participants received a homologous booster dose with the Ad26.COV2.S vaccine (HOB), while the other half of participants had a heterologous vaccination with BNT162b2 COVID-19 vaccine (HEB). The two groups were age- and sex-matched [*p*-value = 0.999 and *p*-value = 0.069, respectively].

The median age of the HOB group was 37 (IQR: 23) years and 50% (5/10) were males. The median BMI was 23.1 (IQR: 5.3) kg/m^2^ and most of them were non-smokers (80%, 8/10). Three participants (30%, 3/10) had comorbidities and six (60%) had allergies. 

The median age of the HEB group was 25 (IQR: 12) years and males were 60% (6/10). The median BMI was 23.6 (IQR: 4.3) kg/m^2^ and 40% (4/10) were smokers. Two (20%) participants had comorbidities and three (30%) had allergies. 

After the booster dose, the most common local AE for both vaccines was pain (60%, 6/10), while the most common systemic AEs were myalgias (40%, 4/10) after the Ad26.COV2.S vaccine and fatigue (50%, 5/10) after the BNT162b2 vaccine. No serious AE was recorded.

### 3.2. Cellular Immune Response

To investigate the TCR repertoires induced by homologous and heterologous COVID-19 vaccination, targeted NGS for the TCRβ locus was performed in two groups (*n* = 10/group). Across all 20 vaccinees’ samples, 1,621,957 templates representing 1,198,341 unique TCRβ CDR3 nucleotide rearrangements were detected, demonstrating the sensitivity of the assay. Of these unique rearrangements, 81.5% were productive, encoding functional proteins. The highest productive Simpson clonality was 0.098, indicating the high diversity (polyclonality) of these repertoires.

**Figure 1:**
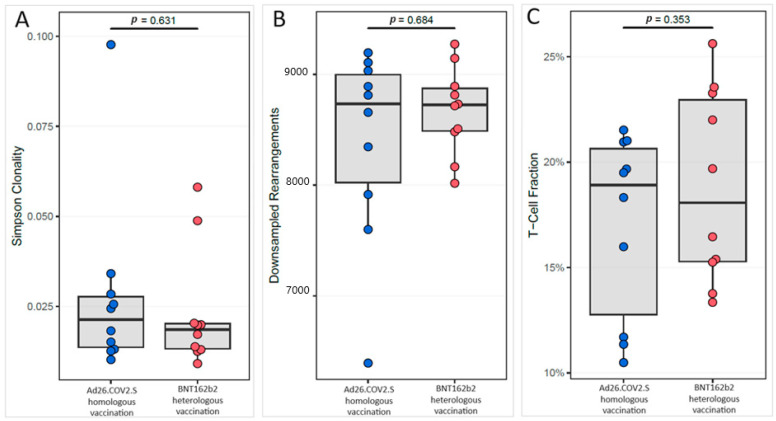
Box plot of T-cell receptor beta metrics reflecting the immune competency (**A**) Simpson clonality; (**B**) Downsampled rearrangements; and (**C**) T-cell fraction in two groups with the Ad26.COV2.S homologous vaccination (blue color) and with the Ad26.COV2.S–BNT162b2 heterologous vaccination (red color). Significant *p*-value was <0.05 using the Mann–Whitney test.

Comparing the two groups, no significant association was found between the median values of Simpson clonality [HOB: 0.021 (IQR: 0.014) vs. HEB: 0.019 (IQR: 0.007)], richness [HOB: 8734.5 (IQR: 973.3) vs. HEB: 8724 (IQR: 383.7)] and T-cell fraction [HOB: 0.19 (IQR: 0.08) vs. HEB: 0.18 (IQR: 0.08)] (Figure 1).

**Figure 2:**
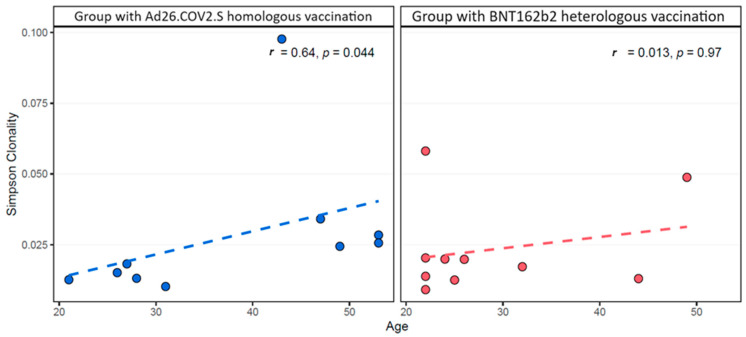
Spearman correlation of Simpson clonality of TCRβ rearrangements with the age of the group with the Ad26.COV2.S homologous vaccination (**left**, blue color) and of group with Ad26.COV2.S–BNT162b2 heterologous vaccination (**right**, red color). Positive correlation was detected in the former group with a statistically significant *p*-value < 0.05.

Correlations between the TCRβ metrics (Simpson clonality, richness and T-cell fraction) and epidemiological data per group showed a significant positive correlation of Simpson clonality with the age of the HOB group (rho = 0.644, *p*-value = 0.04) (Figure 2, Figures S1 and S2).

TCRβ receptor rearrangements which are associated with SARS-CoV-2 were also found using the ImmuneCODE database. All the TCRβ rearrangements values detected in both groups are presented in Table 1. 

No significant differences were detected regarding the comparison of relative frequencies of rearrangements between the two study groups (spike TCRs: HOB 17.8% and HEB 17.1%; non-spike TCRs: HOB 82.2% and 82.9%) (Table 1). However, absolute counts were significantly higher in the HEB compared to HOB group for all investigated rearrangement level metrics; the means (±SD) of total rearrangements were 77515.1 ± 22616.4 vs. 42,675.2 ± 13,610.4 (*p*-value = 0.001), total productive rearrangements (TPRs) were 63,151.8 ± 18,441.5 vs. 34,915.4 ±11,121.6 (*p*-value = 0.001), COVID-19-specific TPRs 522.5 ± 178.0 vs. 298.3 ± 101.1 (*p*-value = 0.003), TPRs specific for SARS-CoV-2 spike protein 90.2 ± 35.1 vs. 52.9 ±19.5 (*p*-value = 0.009) and TPRs specific for SARS-CoV-2 non-spike regions 432.3 ± 144.8 vs. 245.4 ± 83.2 (*p*-value = 0.03). 

**Figure 3:**
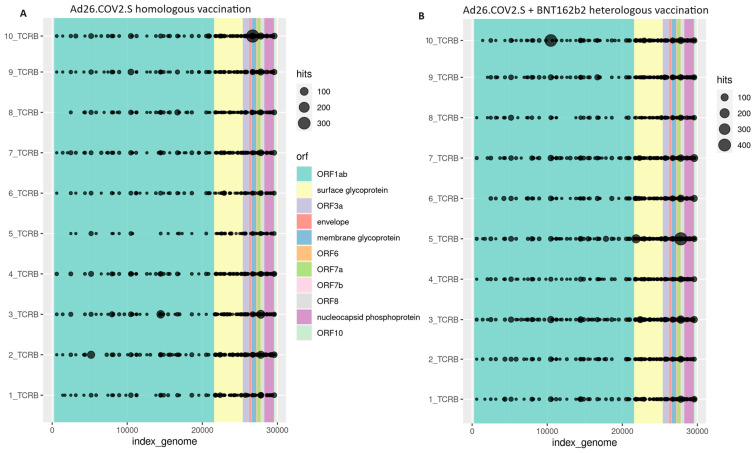
TCRβ rearrangements across the SARS-CoV-2 genome by sample from cohorts of individuals homologously vaccinated with the Ad26.COV2.S COVID-19 vaccine (**A**) and individuals heterologously vaccinated with the Ad26.COV2.S and BNT162b2 COVID-19 vaccines (**B**).

TCRβ rearrangements across the SARS-CoV-2 genome for each individual of both groups are presented in Figure 3. In Samples 1, 4, 6 and 8 from individuals homologously vaccinated with the Ad26.COV2.S COVID-19 vaccine did not detect TCRβ rearrangements against the ORF6 region of SARS-CoV-2. Sample 6 did not also contain TCRβ rearrangement across the ORF8 region. All the other samples carried TCRβ rearrangements across all the regions of the SARS-CoV-2 genome (Figure 3).

An analysis of TCRβ V gene usage showed a wide range of different clonotypes. The most common TRB variable (TRBV) gene clonotypes associated with COVID-19 detected in the group with homologous vaccination were TCRBV27-01 (*n* = 6), TCRBV12-03/12-04 (*n* = 3), TCRBV07-09 (*n* = 1) and TCRBV09-01 (*n* = 1). In this group, one participant had an equal dominant number of the TCRBV27-01 and TCRBV12-03/12-04 clonotypes. In the group with heterologous vaccination, the common TRBV clonotypes were TCRBV27-01 (*n* = 5), TCRBV12-03/12-04 (*n* = 4) and TCRBV09-01 (*n* = 1).

### 3.3. Humoral Immune Response

All participants had detectable TAbs-S titers and were negative for TAbs-N. The HEB cohort had a significant 5.7-fold higher median TAbs-S titer compared with the HOB cohort one month after the booster dose [median (IQR): 10,115.0 (6993.0) U/mL vs. 1781.0 (1314.0) U/mL, *p*-value = 0.001] (Table 2).

## 4. Discussion

In this study, we examined the T-cell repertoires and humoral responses in adults who were immunized with one dose of the Ad26.COV2.S vaccine and were boosted with either the Ad26.COV2.S or BNT162b2 vaccines. Using T-cell receptor beta (TCRβ) sequencing to assess the cellular immune response, we detected in the HEB group an increase in the absolute number of total and COVID-19 related T-cell rearrangements compared to HOB group, which was concordant with the humoral immune responses one month after the boosting. The results of the study indicate that the administration of different COVID-19 vaccines as a booster may provide better protection through enhancement of the T-cell repertoire. This enhancement of the T-cell repertoire probably indicates a better protection against different SARS-CoV-2 variants. That is important for future decisions regarding the type of COVID-19 vaccine to be used as a booster and for better control of future SARS-CoV-2 variants.

The innovation and evolution of next generation sequencing methodologies facilitate the decoding and deep analysis of complex scientific questions. The TCRβ sequencing enables the identification and quantification of antigen-specific T-cell receptors as well as their clonotypes [28,29]. This approach is accurate and can eliminate false negative results of other different methods, for example ELISA, as it is based on deep sequencing with high sensitivity and specificity [12,30]. It can be used for comparative studies of different diseases or patients with specific diseases.

To date, this assay has been used in 27 infectious diseases studies for SARS-CoV-2 [31], Hepatitis B and C [32], Mycobacterium tuberculosis [33], etc., and in 5 vaccine efficacy studies for SARS-CoV-2 [18], influenza [17], glioma [34], etc.

In this study and according to the literature, three metrics for TCR repertoire were used. Simpson clonality and downsampled rearrangements are often used to describe the overall evenness and richness of the TCR repertoire, respectively, which is robust to sequencing depth. The T-cell fraction quantifies the percentage of T cells amongst all nucleated cells in the periphery [23,24,25]. The lack of significant differences in these metrics between the two vaccine groups may be explained by age- and sex-matching.

Alter et al. used this assay to study the cellular response after the first dose of the Ad26.COV2.S vaccine in a cohort similar in size and age of participants to the present study two months after vaccination. Although they also found a wide range of TCRβ rearrangements, the spike-specific T-cell breadth (relative proportion) between vaccinees and convalescent cohorts did not differ significantly [18]. In our study, the spike-specific T-cell rearrangements were significantly elevated in the HEB cohort by absolute count (but not relative proportion). 

Regarding boosting with different COVID-19 vaccines, there is a study testing the T-cell response using an intracellular cytokine staining assay with a 27-color flow cytometry panel after homologous and heterologous COVID-19 vaccination including the Ad26.COV2.S, mRNA-1273 and BNT162b2 vaccines, and the cellular response was found to increase after all vaccine regimens except for the homologous Ad26.COV2.S vaccine regimen [35], similar to our study. 

In the present study, a significant rate of TCRs against non-spike SARS-CoV-2 regions was detected in both groups. Before we included participants in the study, we tested them for SARS-CoV-2 natural infection with nucleocapsid antibody, which is specific to SARS-CoV-2 and does not increase with SARS-CoV-2 immunization. Hence, a possible explanation for the non-spike TCRs is the genetic homology between human coronaviruses (hCoVs) and the T-cell memory in common hCoV pathogens (OC43, HKU1, 229E, NL63). There are limited data on the sequencing and analysis of T-cell repertoires which are developed after COVID-19 immunization; however, the detection of non-spike-specific SARS-CoV-2 TCRβ rearrangements has also been reported in other studies [18,36,37]. Although there are no differences in the HOB and HEB groups regarding the percentage of COVID-19-related TCRs (spike TCRs: HOB 17.8% and HEB 17.1%; non-spike TCRs: HOB 82.2% and 82.9%), the numbers of these TCRs are significantly different between groups for spike (HOB 52.9 and HEB 90.2) and non-spike (HOB 245.4 and HEB 432.3). 

In the present study, TCRβ V gene usage analysis revealed that the TCRBV27-01 clonotype was abundant in both study groups. In another study investigating the differences between patients with severe COVID-19 and healthy controls, they found that the COVID-19 group had more TCRBV5-5, TCRBV6-5, TCRBV7-3, TCRBV7-6, TCRBV7-7, TCRBV7-8, TCRBV10-1 and TCRBV30 clonotypes [38]. Sacco et al. performed the same analysis in children with COVID-19 and multisystem inflammatory syndrome in children (MIS-C) and they found a significant increase in the TRBV11-2 clonotype in children with MIS-C and primarily near the hospitalization period [31]. These differences in the predominance of TRBV clonotypes may be a marker for the specific immunological induction after vaccination or natural infection in adults and children. However, further studies should be performed to elucidate this hypothesis.

Although the total antibodies against wild type SARS-CoV-2 spike protein were detected in all study participants, nevertheless were significantly increased in the HEB group. In accordance with our results, Robins et al. in a study involving approximately five million participants boosted with homologous or heterologous COVID-19 vaccines found that the administration of an mRNA vaccine subsequent to the initial Ad26.COV2.S COVID-19 vaccine dose exhibited more robust antibody responses compared to the use of a homologous boosting approach [17]. They also observed that participants with a heterologous vaccine regimen presented lower severe or critical breakthrough infections than those with homologous vaccination [17]. 

In another study by Atmar et al., SARS-CoV-2 total and neutralizing antibody titers in individuals with primary Ad26.COV2.S COVID-19 vaccination were also significantly higher in heterologous boosters with the BNT162b2 vaccine compared to homologous boosters [18]. These results are in line with the 5.7-fold increase in total SARS-CoV-2 antibodies one month after the heterologous booster presented in the current study which are indicative and could be supportive of the suggested mix-and-match vaccination strategy for pandemic control [19]. 

In this study, no severe AEs were described in line with the findings of other large-scale studies regarding the reactogenicity of Ad26.COV2.S and BNT162b2 COVID-19 primary doses and boosters [4,35,39]. Here, no association was found between the humoral and cellular responses and AEs depending on vaccine regimen, while in other studies with a primary COVID-19 vaccination schedule or after SARS-CoV-2 infection immune response associations with symptoms or AEs have been reported [5,40].

The limitations of this study are the small number of participants and the absence of a control group with healthy unvaccinated individuals or those only infected with SARS-CoV-2. However, TCRβ sequencing is a costly assay and similar studies have around the same number of participants. The strengths of this study include the age- and sex- matching of the HOB and HEB groups, as well as the absence of SARS-CoV-2 infected participants, which allowed the assessment of the immune response induced only by COVID-19 vaccination. 

## 5. Conclusions

The findings of the present study indicated that HEB had increased SARS-CoV-2-related T-cell rearrangements, which was in accordance with the higher humoral responses and possibly conferring longer protection. The utilization of T-cell repertoire sequencing could facilitate the identification and characterization of T-cell responses that are specific to COVID-19, provide valuable information regarding infection and immunization and support the optimization of vaccination strategies.

## Figures and Tables

**Table 1:** Total T-cell receptor rearrangements beta (TCRβ) and COVID-19 specific TCRβ values in 20 participants who were initially immunized with Ad26.COV2.S and were boosted with a homologous (*n* = 10) or heterologous BNT162b2 (*n* = 10) vaccine.

Booster	Samples	Total Rearrangements (Productive and Non-Productive)	Total Productive Rearrangements (TPRs)		COVID-19 TPRs		Spike COVID-19 TPRs		Non-Spike COVID-19 TPRs	
		*n*	*n*	%	*n*	%	*n*	%	*n*	%
**Homologous** **(Ad26.COV2.S)**	1	40,539	32,584	80.4	280	0.86	64	22.9	216	77.1
	2	67,002	54,912	81.9	491	0.89	94	19.1	397	80.9
	3	39,855	31,820	79.8	273	0.86	50	18.3	223	81.7
	4	49,515	39,218	79.2	320	0.82	55	17.2	265	82.8
	5	17,275	14,297	82.8	120	0.84	23	19.2	97	80.8
	6	29,311	24,534	83.7	192	0.78	30	15.6	162	84.4
	7	45,995	37,872	82.3	310	0.82	56	18.1	254	81.9
	8	36,642	30,725	83.9	267	0.87	44	16.5	223	83.5
	9	46,544	37,636	80.9	354	0.94	51	14.4	303	85.6
	10	54,074	45,556	84.3	376	0.83	62	16.5	314	83.5
	Mean (±SD)	42,675.2 (±13,610.4)	34,915.4 (±11,121.6)	81.9 (±1.8)	298.3 (±101.1)	0.85 (±0.1)	52.9 (±19.5)	17.8 (±2.3)	245.4 (±83.2)	82.2 (±2.3)
Heterologous (BNT162b2)	1	76,222	60,980	80.0	478	0.78	83	17.36	395	82.6
	2	72,380	57,184	79.0	430	0.75	68	15.81	362	84.2
	3	118,297	95,538	80.8	903	0.95	163	18.05	740	82.0
	4	55,435	45,147	81.4	385	0.85	68	17.66	317	82.3
	5	104,044	86,987	83.6	668	0.77	112	16.77	556	83.2
	6	70,600	59,007	83.6	430	0.73	76	17.67	354	82.3
	7	71,065	60,089	84.6	591	0.98	90	15.23	501	84.8
	8	40,028	32,497	81.2	249	0.77	36	14.46	213	85.5
	9	74,774	60,613	81.1	529	0.87	83	15.69	446	84.3
	10	92,306	73,476	79.6	562	0.76	123	21.89	439	78.1
	Mean (±SD)	77,515.1 (±22,616.4)	63,151.8 (±18,441.5)	81.5 (±1.9)	522.5 (±178.0)	0.82 (±0.1)	90.2 (±35.1)	17.06 (±2.1)	432.3 (±144.8)	82.9 (±2.1)
*p*-value		**0.001**	**0.001**	0.600	**0.003**	0.351	**0.009**	0.479	**0.003**	0.480

Values refer to the mean ± standard deviation (SD); *p*-value of *t*-test. Statistically significant values are marked in bold.

**Table 2:** Kinetics of total antibodies against SARS-CoV-2 wild type spike protein (TAbs-S) in 20 participants vaccinated for COVID-19.

Vaccine Booster	Tabs-S (U/mL)
**Homologous (Ad26.COV2.S)** ***n* = 10**	1781.0 (1314.0)
**Heterologous (BNT162b2)** ***n* = 10**	10,115.0 (6993.0)
***p*-value**	**0.001**

**Abbreviations:** Values refer to the median ± interquartile range; *p*-value of the Mann–Whitney U test. Statistically significant values are marked in bold.

## Data Availability

All relevant data are within the paper.

## Supplementary Materials

The following supporting information can be downloaded at: https://www.mdpi.com/article/10.3390/pathogens13040284/s1, Figure S1. Spearman correlation of downsampled rearrangements (A) and T-cell fraction (B) metrics of TCRβ rearrangements with the age of the group with the Ad26.COV2.S homologous vaccination (left, blue color) and of the group with the Ad26.COV2.S–BNT162b2 heterologous vaccination (right, red color). A significant *p*-value was <0.05.; Figure S2. Box plot of T-cell receptor beta metrics reflecting the immune competency (A. Simpson clonality; B. Downsampled rearrangements; and C. T-cell fraction) with the sex of the Ad26.COV2.S homologous vaccination (blue color) and BNT162b2 heterologous vaccination (red color) groups. A significant *p*-value was <0.05 using the Mann–Whitney test.

## References

[1] Tatsi E.-B., Filippatos F., Michos A. (2021). SARS-CoV-2 Variants and Effectiveness of Vaccines: A Review of Current Evidence. Epidemiol. Infect..

[2] Tai W., He L., Zhang X., Pu J., Voronin D., Jiang S., Zhou Y., Du L. (2020). Characterization of the Receptor-Binding Domain (RBD) of 2019 Novel Coronavirus: Implication for Development of RBD Protein as a Viral Attachment Inhibitor and Vaccine. Cell. Mol. Immunol..

[3] Cari L., Naghavi Alhosseini M., Mencacci A., Migliorati G., Nocentini G. (2023). Differences in the Expression Levels of SARS-CoV-2 Spike Protein in Cells Treated with MRNA-Based COVID-19 Vaccines: A Study on Vaccines from the Real World. Vaccines.

[4] Sadoff J., Gray G., Vandebosch A., Cárdenas V., Shukarev G., Grinsztejn B., Goepfert P.A., Truyers C., Fennema H., Spiessens B. (2021). Safety and Efficacy of Single-Dose Ad26.COV2.S Vaccine against COVID-19. N. Engl. J. Med..

[5] Michos A., Tatsi E.B., Filippatos F., Dellis C., Koukou D., Efthymiou V., Kastrinelli E., Mantzou A., Syriopoulou V. (2021). Association of Total and Neutralizing SARS-CoV-2 Spike -Receptor Binding Domain Antibodies with Epidemiological and Clinical Characteristics after Immunization with the 1st and 2nd Doses of the BNT162b2 Vaccine. Vaccine.

[6] Tatsi E.B., Filippatos F., Dellis C., Dourdouna M.M., Syriopoulou V., Michos A. (2023). Kinetics of SARS-CoV-2 Spike Antibodies after the Second and Third Dose of the BNT162b2 COVID-19 Vaccine and Association with Epidemiological Characteristics and Breakthrough Infection in a Cohort Study of Healthcare Workers. Microorganisms.

[7] Kanokudom S., Assawakosri S., Suntronwong N., Auphimai C., Nilyanimit P., Vichaiwattana P., Thongmee T., Yorsaeng R., Srimuan D., Thatsanatorn T. (2022). Safety and Immunogenicity of the Third Booster Dose with Inactivated, Viral Vector, and MRNA COVID-19 Vaccines in Fully Immunized Healthy Adults with Inactivated Vaccine. Vaccines.

[8] Tarke A., Potesta M., Varchetta S., Fenoglio D., Iannetta M., Sarmati L., Mele D., Dentone C., Bassetti M., Montesano C. (2022). Early and Polyantigenic CD4 T Cell Responses Correlate with Mild Disease in Acute COVID-19 Donors. Int. J. Mol. Sci..

[9] Jung J.H., Rha M.S., Sa M., Choi H.K., Jeon J.H., Seok H., Park D.W., Park S.H., Jeong H.W., Choi W.S. (2021). SARS-CoV-2-Specific T Cell Memory Is Sustained in COVID-19 Convalescent Patients for 10 Months with Successful Development of Stem Cell-like Memory T Cells. Nat. Commun..

[10] Dourdouna M.M., Tatsi E.B., Syriopoulou V., Michos A. (2023). Evaluation of T Cell Responses with the QuantiFERON SARS-CoV-2 Assay in Individuals with 3 Doses of BNT162b2 Vaccine, SARS-CoV-2 Infection, or Hybrid Immunity. Diagn. Microbiol. Infect. Dis..

[11] Schwarz M., Mzoughi S., Lozano-Ojalvo D., Tan A.T., Bertoletti A., Guccione E. (2022). T Cell Immunity Is Key to the Pandemic Endgame: How to Measure and Monitor It. Curr. Res. Immunol..

[12] Sheridan C. (2021). COVID-19 Testing Turns to T Cells. Nat. Biotechnol..

[13] Rossjohn J., Gras S., Miles J.J., Turner S.J., Godfrey D.I., McCluskey J. (2015). T Cell Antigen Receptor Recognition of Antigen-Presenting Molecules. Annu. Rev. Immunol..

[14] Robins H.S., Campregher P.V., Srivastava S.K., Wacher A., Turtle C.J., Kahsai O., Riddell S.R., Warren E.H., Carlson C.S. (2009). Comprehensive Assessment of T-Cell Receptor Beta-Chain Diversity in Alphabeta T Cells. Blood.

[15] Soto C., Bombardi R.G., Kozhevnikov M., Sinkovits R.S., Chen E.C., Branchizio A., Kose N., Day S.B., Pilkinton M., Gujral M. (2020). High Frequency of Shared Clonotypes in Human T Cell Receptor Repertoires. Cell Rep..

[16] DeWitt W.S., Emerson R.O., Lindau P., Vignali M., Snyder T.M., Desmarais C., Sanders C., Utsugi H., Warren E.H., McElrath J. (2015). Dynamics of the Cytotoxic T Cell Response to a Model of Acute Viral Infection. J. Virol..

[17] Herati R.S., Muselman A., Vella L., Bengsch B., Parkhouse K., Del Alcazar D., Kotzin J., Doyle S.A., Tebas P., Hensley S.E. (2017). Successive Annual Influenza Vaccination Induces a Recurrent Oligoclonotypic Memory Response in Circulating T Follicular Helper Cells. Sci. Immunol..

[18] Alter G., Yu J., Liu J., Chandrashekar A., Borducchi E.N., Tostanoski L.H., McMahan K., Jacob-Dolan C., Martinez D.R., Chang A. (2021). Immunogenicity of Ad26.COV2.S Vaccine against SARS-CoV-2 Variants in Humans. Nature.

[19] Dykema A.G., Zhang B., Woldemeskel B.A., Garliss C.C., Rashid R., Westlake T., Zhang L., Zhang J., Cheung L.S., Caushi J.X. (2022). SARS-CoV-2 Vaccination Diversifies the CD4+ Spike-Reactive T Cell Repertoire in Patients with Prior SARS-CoV-2 Infection. eBioMedicine.

[20] Yin Q., Luo W., Mallajosyula V., Bo Y., Guo J., Xie J., Sun M., Verma R., Li C., Constantz C.M. (2023). A TLR7-Nanoparticle Adjuvant Promotes a Broad Immune Response against Heterologous Strains of Influenza and SARS-CoV-2. Nat. Mater..

[21] Carlson C.S., Emerson R.O., Sherwood A.M., Desmarais C., Chung M.W., Parsons J.M., Steen M.S., LaMadrid-Herrmannsfeldt M.A., Williamson D.W., Livingston R.J. (2013). Using Synthetic Templates to Design an Unbiased Multiplex PCR Assay. Nat. Commun..

[22] Robins H., Desmarais C., Matthis J., Livingston R., Andriesen J., Reijonen H., Carlson C., Nepom G., Yee C., Cerosaletti K. (2012). Ultra-Sensitive Detection of Rare T Cell Clones. J. Immunol. Methods.

[23] Chiffelle J., Genolet R., Perez M.A., Coukos G., Zoete V., Harari A. (2020). T-Cell Repertoire Analysis and Metrics of Diversity and Clonality. Curr. Opin. Biotechnol..

[24] Kim B.R., Shin J., Guevarra R.B., Lee J.H., Kim D.W., Seol K.H., Lee J.H., Kim H.B., Isaacson R.E. (2017). Deciphering Diversity Indices for a Better Understanding of Microbial Communities. J. Microbiol. Biotechnol..

[25] Reuben A., Zhang J., Chiou S.H., Gittelman R.M., Li J., Lee W.C., Fujimoto J., Behrens C., Liu X., Wang F. (2020). Comprehensive T Cell Repertoire Characterization of Non-Small Cell Lung Cancer. Nat. Commun..

[26] Nolan S., Vignali M., Klinger M., Dines J.N., Kaplan I.M., Svejnoha E., Craft T., Boland K., Pesesky M., Gittelman R.M. (2020). A Large-Scale Database of T-Cell Receptor Beta (TCRβ) Sequences and Binding Associations from Natural and Synthetic Exposure to SARS-CoV-2. Res. Sq..

[27] Dines J.N., Manley T.J., Svejnoha E., Simmons H.M., Taniguchi R., Klinger M., Baldo L., Robins H. (2020). The ImmuneRACE Study: A Prospective Multicohort Study of Immune Response Action to COVID-19 Events with the ImmuneCODE^TM^ Open Access Database. medRxiv.

[28] Warren R.L., Freeman J.D., Zeng T., Choe G., Munro S., Moore R., Webb J.R., Holt R.A. (2011). Exhaustive T-Cell Repertoire Sequencing of Human Peripheral Blood Samples Reveals Signatures of Antigen Selection and a Directly Measured Repertoire Size of at Least 1 Million Clonotypes. Genome Res..

[29] Shen Y., Voigt A., Leng X., Rodriguez A.A., Nguyen C.Q. (2023). A Current and Future Perspective on T Cell Receptor Repertoire Profiling. Front. Genet..

[30] Freeman J.D., Warren R.L., Webb J.R., Nelson B.H., Holt R.A. (2009). Profiling the T-Cell Receptor Beta-Chain Repertoire by Massively Parallel Sequencing. Genome Res..

[31] Sacco K., Castagnoli R., Vakkilainen S., Liu C., Delmonte O.M., Oguz C., Kaplan I.M., Alehashemi S., Burbelo P.D., Bhuyan F. (2022). Immunopathological Signatures in Multisystem Inflammatory Syndrome in Children and Pediatric COVID-19. Nat. Med..

[32] Mazouz S., Boisvert M., Abdel-Hakeem M.S., Khedr O., Bruneau J., Shoukry N.H. (2021). Expansion of Unique Hepatitis C Virus–Specific Public CD8+ T Cell Clonotypes during Acute Infection and Reinfection. J. Immunol..

[33] Musvosvi M., Huang H., Wang C., Xia Q., Rozot V., Krishnan A., Acs P., Cheruku A., Obermoser G., Leslie A. (2023). T Cell Receptor Repertoires Associated with Control and Disease Progression Following Mycobacterium Tuberculosis Infection. Nat. Med..

[34] Platten M., Bunse L., Wick A., Bunse T., Le Cornet L., Harting I., Sahm F., Sanghvi K., Tan C.L., Poschke I. (2021). A Vaccine Targeting Mutant IDH1 in Newly Diagnosed Glioma. Nature.

[35] Atmar R.L., Lyke K.E., Deming M.E., Jackson L.A., Branche A.R., El Sahly H.M., Rostad C.A., Martin J.M., Johnston C., Rupp R.E. (2022). Homologous and Heterologous Covid-19 Booster Vaccinations. N. Engl. J. Med..

[36] Soni M., Migliori E., Fu J., Assal A., Chan H.T., Pan J., Khatiwada P., Ciubotariu R., May M.S., Pereira M. (2023). The Prospect of Universal Coronavirus Immunity: A Characterization of Reciprocal and Non-Reciprocal T Cell Responses against SARS-CoV2 and Common Human Coronaviruses. Biorxiv Prepr. Serv. Biol..

[37] Dykema A.G., Zhang B., Woldemeskel B.A., Garliss C.C., Cheung L.S., Choudhury D., Zhang J., Aparicio L., Bom S., Rashid R. (2021). Functional Characterization of CD4+ T Cell Receptors Crossreactive for SARS-CoV-2 and Endemic Coronaviruses. J. Clin. Investig..

[38] Corpas M., de Mendoza C., Moreno-Torres V., Pintos I., Seoane P., Perkins J.R., Ranea J.A.G., Fatumo S., Korcsmaros T., Martín-Villa J.M. (2023). Genetic Signature Detected in T Cell Receptors from Patients with Severe COVID-19. iScience.

[39] Polack F.P., Thomas S.J., Kitchin N., Absalon J., Gurtman A., Lockhart S., Perez J.L., Pérez Marc G., Moreira E.D., Zerbini C. (2020). Safety and Efficacy of the BNT162b2 MRNA Covid-19 Vaccine. N. Engl. J. Med..

[40] Elyanow R., Snyder T.M., Dalai S.C., Gittelman R.M., Boonyaratanakornkit J., Wald A., Selke S., Wener M.H., Morishima C., Greninger A.L. (2022). T Cell Receptor Sequencing Identifies Prior SARS-CoV-2 Infection and Correlates with Neutralizing Antibodies and Disease Severity. JCI Insight.

