# Supplementary material for: Comparative Study of T-Cell Repertoires after COVID-19 Immunization with Homologous or Heterologous Vaccine Booster
Source: Pathogens. 2024 Mar 27;13(4):284. doi: 10.3390/pathogens13040284 (PMC11054887; doi:10.3390/pathogens13040284)
Supplement: Supplementary file 1 [file pathogens-13-00284-s001.zip › pathogens-2864247-supplementary.pdf]

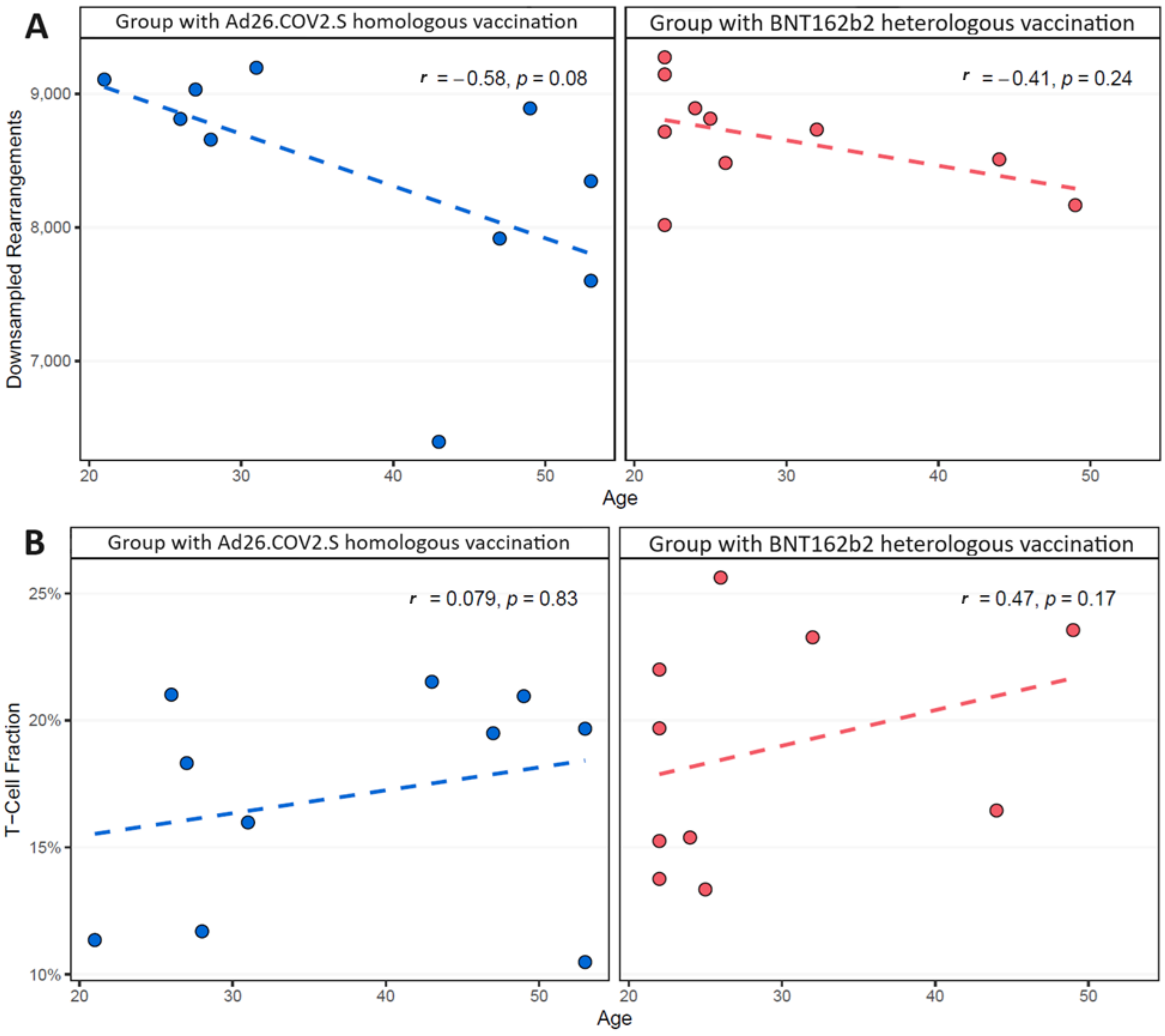

Figure S1. Spearman correlation of Downsampled Rearrangements (A) and T-Cell Fraction (B) metrics of TCRβ rearrangements with age of group with Ad26.COV2.S homologous vaccination (left, blue colour) and of group with Ad26.COV2.S - BNT162b2 heterologous vaccination (right, red colour). Significant *p*-value was <0.05.

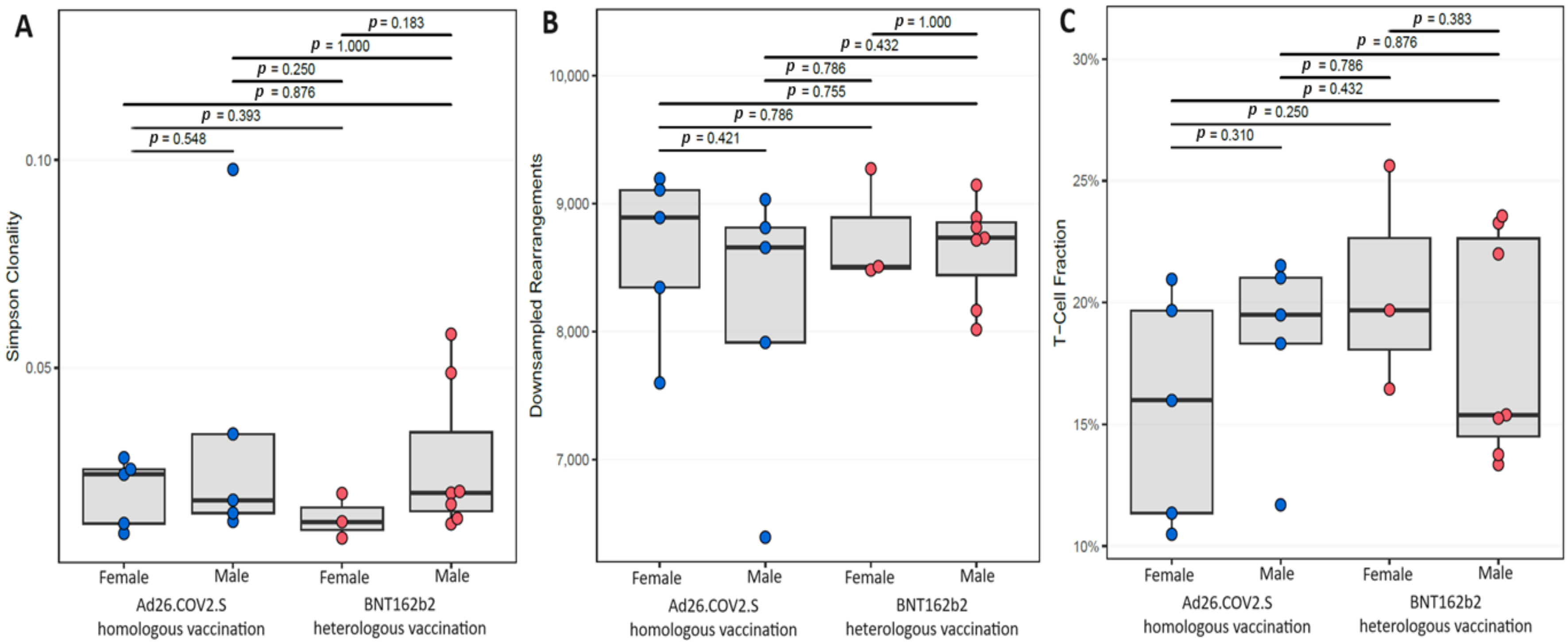

Figure S2. Box plot of T-cell receptor beta metrics reflecting the immune competency (A. Simpson Clonality, B. Downsampled rearrangements and C. T-cell fraction) with sex of Ad26.COV2.S homologous vaccination (blue colour) and BNT162b2 heterologous vaccination (red colour) groups. Significant *p*-value was <0.05 using Mann–Whitney test.
